# A Novel Deep Learning-Based Black Fungus Disease Identification Using Modified Hybrid Learning Methodology

**DOI:** 10.1155/2022/4352730

**Published:** 2022-01-27

**Authors:** S. Karthikeyan, G. Ramkumar, S. Aravindkumar, M. Tamilselvi, S Ramesh, A. Ranjith

**Affiliations:** ^1^Department of Electronics and Communication Engineering, Sathyabama Institute of Science and Technology, Chennai, Tamil Nadu, India; ^2^Department of Electronics and Communication Engineering, Saveetha School of Engineering, Chennai, Tamil Nadu, India; ^3^Department of Information Technology, Rajalakshmi Engineering College, Chennai, Tamil Nadu, India; ^4^Department of Mechatronics Engineering, T. S. Srinivasan Centre for Polytechnic College and Advanced Training, Chennai, Tamil Nadu, India; ^5^Department of Electronics and Communication Engineering, St. Mother Theresa College of Engineering, Vagaikulam-628102, Tamil Nadu, India; ^6^Department of Electrical, Electronics and Communication Engineering, St. Joseph University in Tanzania, Tanzania

## Abstract

Currently, countries across the world are suffering from a prominent viral infection called COVID-19. Most countries are still facing several issues due to this disease, which has resulted in several fatalities. The first COVID-19 wave caused devastation across the world owing to its virulence and led to a massive loss in human lives, impacting the country's economy drastically. A dangerous disease called mucormycosis was discovered worldwide during the second COVID-19 wave, in 2021, which lasted from April to July. The mucormycosis disease is commonly known as “black fungus,” which belongs to the fungus family Mucorales. It is usually a rare disease, but the level of destruction caused by the disease is vast and unpredictable. This disease mainly targets people already suffering from other diseases and consuming heavy medication to counter the disease they are suffering from. This is because of the reduction in antibodies in the affected people. Therefore, the patient's body does not have the ability to act against fungus-oriented infections. This black fungus is more commonly identified in patients with coronavirus disease in certain country. The condition frequently manifests on skin, but it can also harm organs such as eyes and brain. This study intends to design a modified neural network logic for an artificial intelligence (AI) strategy with learning principles, called a hybrid learning-based neural network classifier (HLNNC). The proposed method is based on well-known techniques such as convolutional neural network (CNN) and support vector machine (SVM). This article discusses a dataset containing several eye photographs of patients with and without black fungus infection. These images were collected from the real-time records of people afflicted with COVID followed by the black fungus. This proposed HLNNC scheme identifies the black fungus disease based on the following image processing procedures: image acquisition, preprocessing, feature extraction, and classification; these procedures were performed considering the dataset training and testing principles with proper performance analysis. The results of the procedure are provided in a graphical format with the precise specification, and the efficacy of the proposed method is established.

## 1. Introduction 

In 2019, a dangerous disease known as COVID-19, caused by the novel coronavirus, spread globally, causing severe destruction of human lives and severely impacting global economic growth. During the second wave of the COVID-19 disease, black fungus became a pressing issue in patients afflicted by the novel coronavirus. Because steroid-oriented drugs are consumed to minimize the effect of COVID-19 infection, the immune system of the patients is highly compromised, leading to the rapid spread of such fungal disease. In particular, the disease spread ratio is higher in India than in other countries. Steroids reduce adaptive immunity by lowering respiratory disorders and alleviating the body's natural immunological function, to prevent it from targeting healthy tissues. These patients were highly susceptible to black fungus infection, even after the second wave abated, and the national coronavirus task force declared health alert [1, 2]. The general term of black fungus, which is a rare disease, is mucormycosis. In particular, prior to the global outbreak of coronavirus, the unusual fungal disease afflicted many patients in India, with the disease frequency reported being approximately 70 to 75 times higher than that in the remainder of the world in India [3, 4].

Black fungus is usually observed in persons who have recovered from coronavirus infection and have comorbidities, such as diabetes, kidney disease, heart disease, or other cancer-related diseases. The symptoms of this black fungus infection are similar to those of coronavirus and common flu. If left unaddressed, the disease spreads from the nose to the eye region and brain, resulting in life-threatening problems. The state of Maharashtra (Western), which includes Mumbai City, has documented approximately 2000 black fungus cases and eight deaths due to mucormycosis. The purpose of this study was to examine the black fungus disease, its characteristics, and the method of treatment associated with mucormycosis in coronavirus patients [5, 6].

## 2. Black Fungus

Black fungus or mucormycosis is a rare fungal infection caused by a fungus called micromycetes, which is prevalent in ground and rotting plants. The head of “Westmead Institute for Medical Research,” Julie Djordjevic, analyzing the fungus-oriented diseases, referred to this fungi as environmental ground rot. It is most prevalent in individuals consuming medicines for health complications that impair their ability to resist external infections. The sinus or lung(s) of these patients become infected after inhaling fungal particles from the atmosphere. Additionally, the fungi can affect the brain, skin, and kidneys. Humans can become ill if they inhale or consume certain natural fungal sources, and the fungi can also penetrate the skin via cuts or open sores. The fungi are highly adept at self-replication, reproduce asexually, and are highly aerodynamic, replicating into trillions. This disease affects the regions such as sinus, brain area, and lungs and can be fatal in people with diabetes or even in ordinary people who have significantly less immunity in the body, such as people with cancer or with HIV disease. This disease mainly targets people who already have such complications and consume steroid-based medicines for their illness [7].

**Figure 1:**
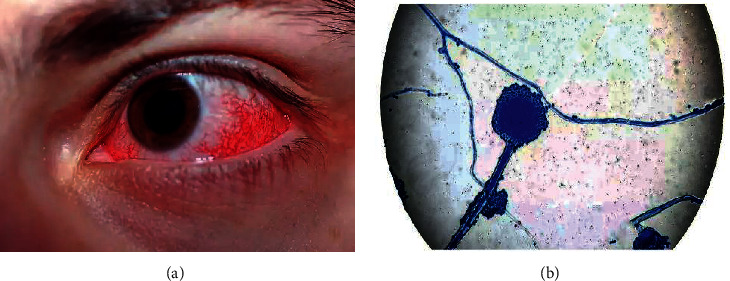
(a) Infected eye with redness indication [6] and (b) mucormycosis.

According to specialists, as a severe fungal disease spreads throughout human blood, it can also harm their eyes. As a result, the eyesight may diminish considerably. A few of the primary indications for this black fungus infection include reddishness in the eyes.  Figure 1(a) illustrates the perception of black fungus eye infection in the preliminary stage [7], and Figure 1(b) illustrates the basic structural overview of mucormycosis with reference to [8, 9].

### 2.1. Dataset

**Figure 2:**
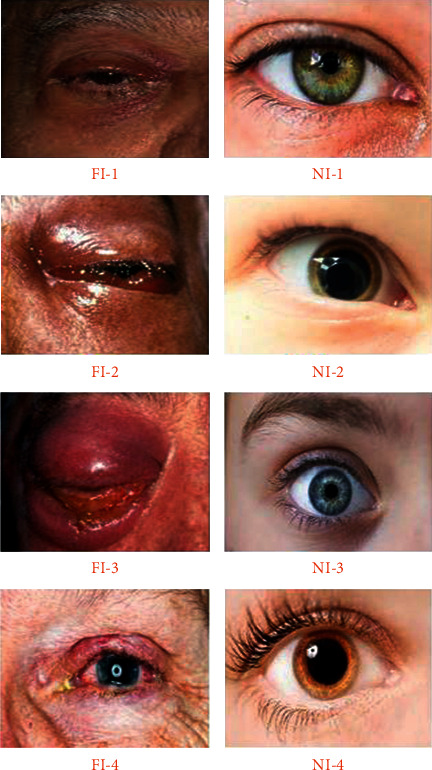
Dataset image samples of both normal fungus and normal images.

Black fungus disease was accurately identified in this study using a custom-defined dataset. Because there is no available dataset for black fungus images, it is necessary to prepare the dataset independently with proper images that include affected and nonaffected cases. This system accumulated black fungus image data in such a way that was machine comprehensible. Thus, the machine can easily understand and predict normal and affected images. The procedure was performed using the proposed deep learning strategy called the hybrid learning-based neural network classifier (HLNNC). This method was applied to the dataset, and the samples were trained accordingly; this process is helpful in creating a standard learning model to predict black fungus disease in a timely and easy manner.  Figure 2 illustrates the dataset sample images of the images that are unaffected and those affected by black fungus, in a graphical format. The declaration labels FI-1, FI-2, FI-3, and FI-4 indicate the eye images affected by black fungus disease, in which the term FI indicates the fungus image (FI). The term NI indicates the normal image without fungal infection.

### 2.2. Motivations of This Disease Prediction

According to the Global Health Survey of 2021, the second coronavirus wave led to massive destruction globally. Individuals contract mucormycosis from the fungi in their surroundings. For instance, diseases of the lungs or sinuses may occur because of inhaling fungi. People with health complications or those using medications that impair the body's natural immune capacity to fight infections and illness are more susceptible to mucormycosis.

All of these factors led to the point in mid-2021, during which most people who contract coronavirus also contracted black fungus as a side effect of the steroid-related medicines used to combat the viral infection. These factors have had a significant impact on the mind and have compelled us to develop a new methodology to intelligently predict the black fungus disease using deep learning algorithms. This study introduced a new hybrid learning algorithm called HLNNC to predict black fungus diseases using regular eye images. The prediction accuracy of the proposed approach is satisfactory, and corresponding proof of this method's accuracy has been demonstrated.  Table 1 presents the number of mucormycosis fungal infections (black fungus) in different states of India. The number of cases crosses 9, 000, and the details are listed in Table 1 [10].

Table 2 shows the shortage of the medication for black fungus (amphotericin B) across states in India [11].

#### 2.2.1. Major Objectives

The following are the primary objectives of the proposed hybrid learning strategy for predicting black fungus:

- Design a new hybrid algorithm to predict the black fungus using a custom-defined real-time dataset
- Provide support to physicians during the pandemic to analyze the disease using machine support and provide faster assistance to people with a maximum accuracy ratio
- Predict the black fungus accurately using the most common conventional algorithms, such as convolutional neural networks and support vector machine classifiers
- Provide robust prediction logic to significantly conserve time in treating people during this critical period

The aforementioned objectives were accomplished by applying these modified learning principles, and the predicted accuracy ratio of the proposed scheme was sufficient for identifying black fungus disease in a real-time context. This system can be suitably adapted in hospitals to identify fungal diseases accurately with the help of computer-aided digital image processing design. The results section provides proper proof for its efficacy.

## 3. Related Study

Mucormycosis is a blanket word that refers to any fungal infection caused by any Zygomycetes genus [12]. Another synonymous word used in medical and layman terminology is phycomycosis. Mucormycosis is a potentially lethal disease caused by various fungi present in the soil and environment. Although these fungal infections are generally uncommon, they occur in individuals who are severely disabled and also in groups of people who have been injured. These injuries may occur in the event of natural catastrophes, including tsunamis, hurricanes, earthquakes, or tornadoes, when polluted soil and water can be ingested, entrenched in wounds, or driven into the skin, mouth, eyes, and nose by the force of water, soil, or wind pressure. The disease is not contagious [12]. Mucormycosis infections were discovered in a cluster of individuals who initially survived terrible storms that devastated Joplin, Missouri, on May 23, 2011. Thirteen cases were confirmed, all of which included individuals who had severe wounds, such as fractures, numerous wounds, penetrating injuries, and blunt trauma. Ten individuals required intensive care, and five succumbed to their injuries. Because most mucormycosis infections are caused by a single Zygomycetes family member (family Mucoraceae), many doctors now refer to the condition as mucormycosis rather than zygomycosis, the more “generic” name. The popular press has coined titles such as “Black Death” and “Zombie illness” to depict this fungal infection; however, these terms rarely assist the public in comprehending the disease. Such terminology may create misconceptions among patients, their families, and the general public [12].

While COVID-19 continues to wreak havoc on the lives of many individuals worldwide, a second pandemic, “black fungus,” has emerged, robbing individuals of their lives, particularly those recovering from the coronavirus [9]. Furthermore, sentiment research on social media can ascertain popular attitudes toward such pandemics. Thus, this research aims to conduct sentiment analysis on public perceptions of black fungus during the COVID-19 epidemic. An SVM model was created with an average AUC of 82.75 percent for classifying user moods into anger, fear, joy, and sadness [9]. Subsequently, this SVM was utilized to monitor the classification of public tweets (*n* = 6477) mentioning COVID-19 and black fungus. This study found that public opinions of black fungus during the COVID-19 pandemic were divided into sad (*n* = 2370, 36.59 percent), joy (*n* = 2095, 32.34 percent), fear (*n* = 1914, 29.55 percent), and wrath (*n* = 98, 1.51 percent). Additionally, this study examined the public attitudes of several crucial topics (such as education, lockdown, hospital, oxygen, quarantine, and vaccine) and discovered that public perceptions varied. For instance, while the majority of people reported anxiety on social media regarding education, hospitals, and vaccines, some expressed joy regarding education, hospitals, vaccines, and oxygen [13].

## 4. System Methodologies

The black fungus disease is identified via several investigation procedures such as computed tomography (CT) scans, medical resonance imaging (MRI) scans, and cell biopsy tests. However, all these investigation procedures are expensive, and ordinary people cannot afford these expensive tests; therefore, this study introduced a new prediction algorithm with proper integration of traditional learning algorithms such as convolutional neural network and support vector machine, which is termed as a hybrid learning-based neural network classifier (HLNNC). This algorithm follows the following principles to identify black fungus diseases, such as image acquisition, image preprocessing, extraction of image features, classification, and accuracy estimations. The aforementioned procedures were followed to predict black fungus disease based on real-time dataset images. To avoid the unexpected costs that may be incurred in predicting the disease, a novel algorithm called HLNNC was designed using regular digital eye images. The following summary illustrates the principles followed in processing digital images in a proper way.

### 4.1. Image Acquisition and Preprocessing

A digital image is considered a two-dimensional depiction of a picture comprising a limited collection of visual quantities known as image pixels or bytes. In digital image processing and artificial intelligence perception, image acquisition refers to obtaining an image from a source, typically a mechanical system such as digital cameras or scanners [14]. This is perhaps the most critical stage in the process chain, considering that the machine cannot perform any operations without an image. Image preprocessing is the procedure of formatting digital images before their use in machine training and validation. This may encompass, but is not restricted to scaling, orientation, and color corrections. These actions do not add to the object's relevant information, but rather subtract from if unpredictability is used as an information metric. The purpose of digital image preprocessing is to improve the image content by suppressing undesirable abnormalities and enhancing specific visual properties that are necessary for subsequent processing and decision-making tasks. The proposed learning strategy, called the hybrid learning-based neural network classifier, acquires the real-time dataset from different patients and manipulates it according to the digital image processing principles.  Algorithm 1 illustrates the steps involved in image acquisition and preprocessing, presented with proper pseudocode specifications.

### 4.2. Extraction of Image Features

The process of extracting the black fungus dataset image features is a subset that reduces the dimensionality of the preprocessed digital images. It divides and reduces the original collection of raw information into much more accessible image categories, including normal and affected images. These image characteristics are simple to handle, even while accurately and uniquely describing the entire original image. Extracting the image features from the input dataset enhances the accuracy of the learned modeling techniques. By deleting unnecessary information, this stage of the standard outline decreases the complexity of the information and naturally speeds up training and interpretation. The purpose of extracting digital image features is to reduce the number of characteristics in a black fungus dataset by generating new features from the traditional procedures of CNN and SVM. These newly condensed characteristics should also be capable of summarizing the majority of the data present in the initial feature set. The feature selection process is the most critical concern in the feature extraction process, in which the image is finely filtered. Filtration-assisted feature selection approaches assess the relationship or correlation among digital image input parameters, which may then be filtered to identify the critical and relevant features of the corresponding image. The measured variables for selecting features must always be selected explicitly about the outputs, inputs, or standard response variables' data formats. This feature selection process identifies the background and foreground features and extracts the foreground features of the black fungus image to attain better accuracy in an outcome.  Algorithm 2 illustrates the feature extraction process flow in detail with proper pseudocode specifications.

### 4.3. Classification

**Figure 3:**
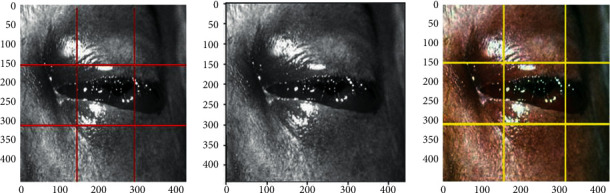
Image variations used for classification.

Classification is a method of categorizing digital images depending on their properties and other features such as object's contours, pixel strength, and variation in image pixel intensities. Following this, the classification system will attempt to comprehend these features. We examine the three mentioned images (Figure 3), which pertain to a specific person, but they differ in various ways, including image color, eye orientation, backdrop color, eyeball color, and surrounding region. The most challenging aspect of interacting with images is the unpredictability of such qualities. The black fungus appears identical to the human eye; however, once transformed to knowledge, it could be difficult to discern a pattern among various images. The variations identified over the images in the following figure are illustrated correctly with the marked regions, and based on such classification methodologies, many complicated issues are resolved perfectly. The proposed hybrid learning-based neural network classifier is a type of deep learning-assisted neural network scheme, in which it is considered that the CNN is an artificial intelligence-enabled machine learning system that can also accept an image as input, ascribe relevance such as trainable weights and biases to different facets in the input image, and distinguish them.  Algorithm 3 illustrates the classification process flow of the proposed HLNNC approach in detail with proper pseudocode specifications.

The three processes mentioned in the algorithms are collectively referred to as the proposed hybrid learning-based neural network classifier. The real-time processing ability of the proposed approach is illustrated clearly over the results section in a transparent manner with graphical proofs.

## 5. Result and Discussion

**Figure 4:**
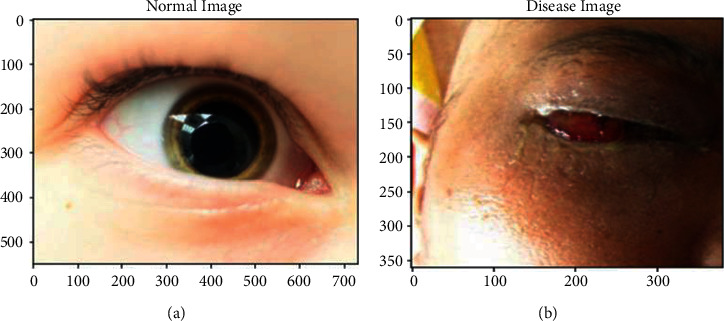
(a) Normal image and (b) diseased image.

A new methodology of HLNNC is designed to predict the black fungus disease using artificial intelligence, and this approach effectively identifies the disease using predictive logic with a high accuracy rate of 99.5%. The proposed approach is implemented using the open-source programming assistance called Python, in which the Jupyter Notebook is a tool to develop the codes to predict the black fungus infection. The resulting scenarios are appropriate and intelligent for identifying the disease using real-time dataset-based training norms. The input dataset was trained using several procedures, which has been clearly elucidated in Section 3.  Figure 4 illustrates the dataset's normal and diseased image views selected for processing. It is just a sample of selection shown to the user perception to prove that the working flow is acceptable.

**Figure 5:**
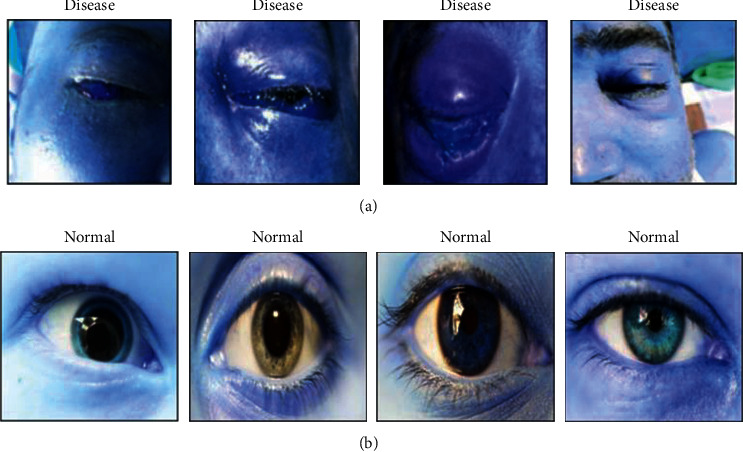
(a) Preprocessed normal images and (b) preprocessed disease images.

Figure 5 illustrates the image reprocessing view of the proposed approach, in which it processes both the affected and standard images in a transparent manner.

**Figure 6:**
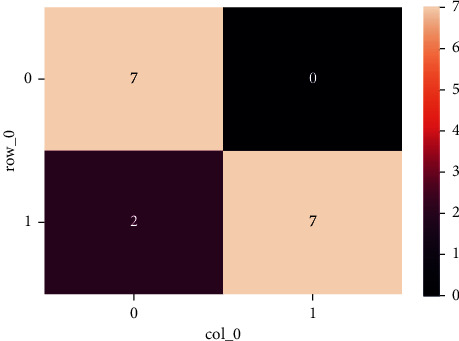
Confusion matrix.

Figure 6 illustrates the confusion matrix of the proposed approach, in which it shows the summary of the predicted outcomes for a classification scenario. The proportion of accurate and inaccurate predictions is reported and divided by category using score metrics. This is the confusion matrix, in which confusion prevails while making predictions.

**Figure 7:**
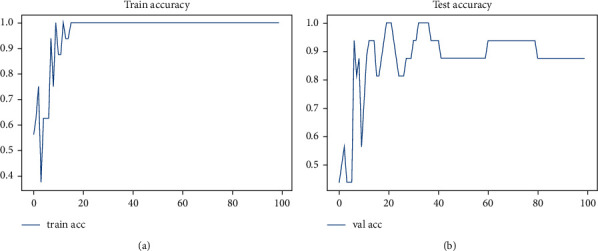
(a) Training accuracy and (b) testing accuracy.

Figure 7 illustrates the training and testing accuracy ratio of the proposed approach, which is evaluated by processing the total number of trained samples and the randomly selected testing or validation inputs.

**Figure 8:**
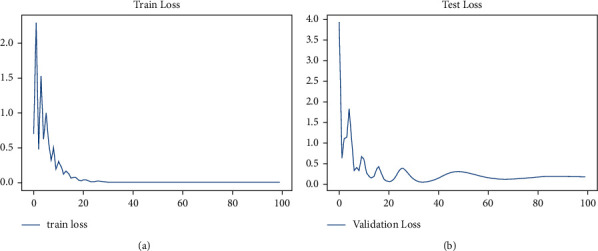
(a) Training loss ratio and (b) testing loss ratio.

Figure 8 illustrates the training and testing loss ratio of the proposed approach, evaluated by processing the total number of trained samples and the randomly selected testing or validation inputs.

**Figure 9:**
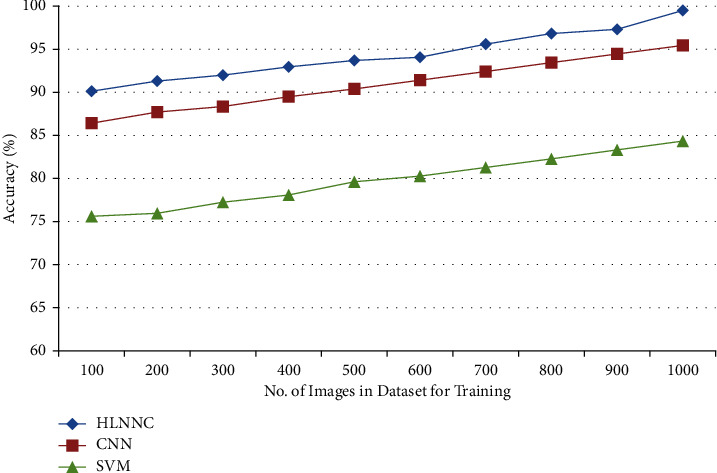
Accuracy evaluation.

Figure 9 shows the proposed HLNNC accuracy ratio, in which it is cross-validated with the conventional classification algorithms such as CNN and SVM. The resulting proof illustrates the efficiency of the proposed approach in a graphical manner.

## 6. Conclusion and Future Scope

Mucormycosis is especially prevalent in patients treated with steroids and similar anti-inflammatory medications for coronavirus. While no significant epidemic has been reported, the federal coronavirus expert panel released a statement. The Black Fungus Infections Research Council and Clinical Infections Society gathered evidence for significant research. This is critical to handle this matter seriously because people from around the globe have been affected psychologically and financially, and it should not further affect the overall quality of life. Additional studies on this subject will aid in eliminating mucormycosis on a global scale. This study provides a logical evaluation of the black fungus disease prediction concerning the proposed hybrid profound learning logic called a hybrid learning-based neural network classifier. This classifier efficiently identifies the black fungus disease without expensive investigations, such as MRI or CT. Apart from these issues, the proposed hybrid logic provides the highest accuracy ratio of approximately 99.5%, which is significant enough to trust the designed model, and this has been supported by the evidence provided in the figures presented in the results section.

In the future, the work can be further enhanced by adding time-constraint algorithms, such as polynomial-time analyzers, to improve the proposed approach timing efficiency, which needs to be improved slightly in the training phase [15].

## Figures and Tables

**Algorithm 1:**
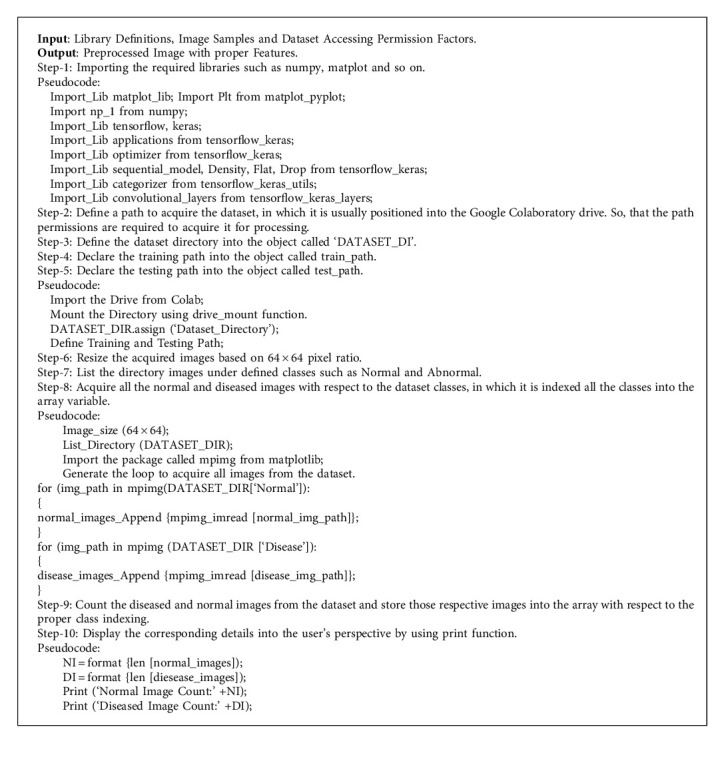
Image acquisition and preprocessing.

**Algorithm 2:**
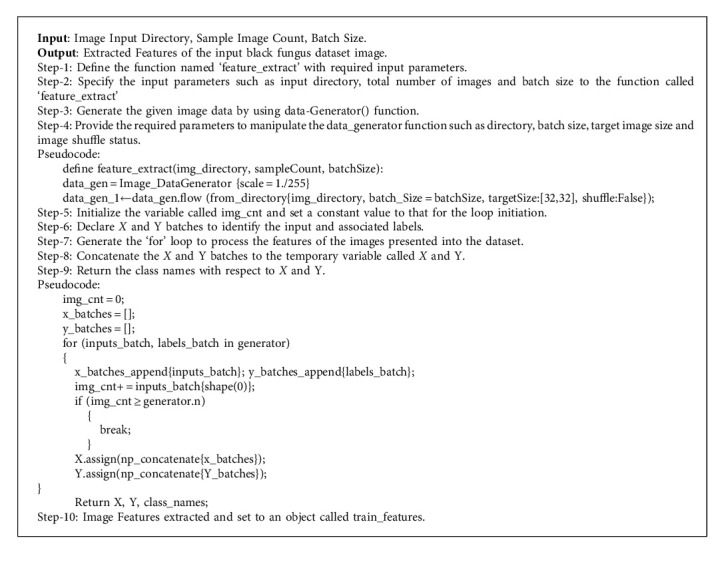
Extracting image features.

**Algorithm 3:**
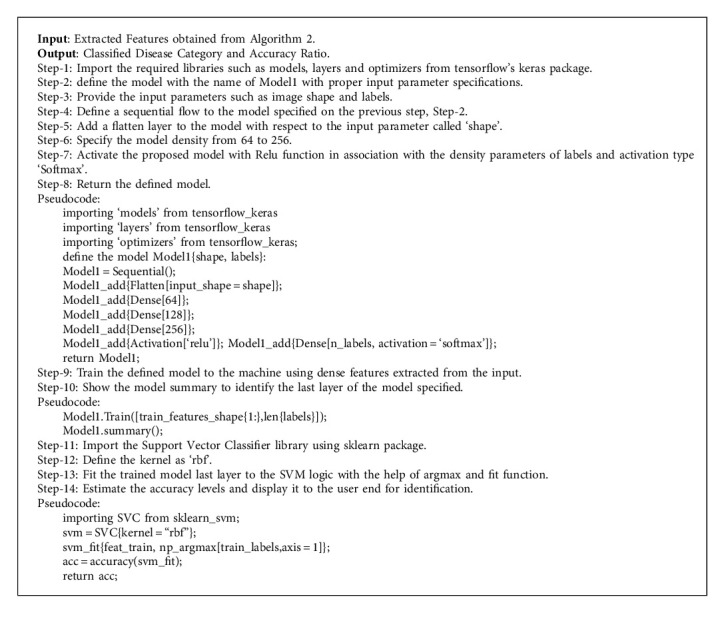
Classification.

**Table 1:** Cases of mucormycosis in India [10].

State	Number of mucormycosis cases
Gujarat	2281
Maharashtra	2000
Andhra Pradesh	910
Madhya Pradesh	720
Rajasthan	700
Karnataka	500
Telangana	350
Haryana	250
Delhi	197
Uttar Pradesh	112
Punjab	95
Chhattisgarh	87
Bihar	56
Tamil Nadu	40
Kerala	36

**Table 2:** Requirement of amphotericin [11].

State	Patients under treatment	No. of vials required per day	Total no. of vials needed for treatment (in lakhs)
Gujarat	2859	17154	7.14
Maharashtra	2770	16620	6.93
Andhra Pradesh	766	4596	1,92
Madhya Pradesh	752	4512	1.88
Telangana	744	4464	1.86
Uttar Pradesh	701	4206	1.75
Rajasthan	492	2952	1.23
Karnataka	481	2886	1.20
Haryana	436	2616	1.09
Tamil Nadu	236	1416	0.59
Bihar	215	1290	0.54
Punjab	141	846	0.35
Uttarakhand	124	744	0.31
Delhi	119	714	0.30
Chhattisgarh	103	618	0.26
Others	184	1104	0.46

## Data Availability

The data used to support the findings of this study are included within the article.
